# Cytogenetically Balanced Reciprocal Translocation Could Hide Molecular Genomic Unbalances: Implications for Foetal Phenotype Correlation

**DOI:** 10.3390/diagnostics14161732

**Published:** 2024-08-09

**Authors:** Nicoletta Villa, Serena Redaelli, Stefania Farina, Elena Sala, Francesca Crosti, Sabrina Cozzolino, Maria Verderio, Leda Dalprà, Gaia Roversi, Angela Bentivegna, Giovanni Cazzaniga, Marialuisa Lavitrano, Donatella Conconi

**Affiliations:** 1 UC Medical Genetics, Fondazione IRCCS San Gerardo dei Tintori, 20900 Monza, Italy; nicoletta.villa@irccs-sangerardo.it (N.V.); s.farina1@campus.unimib.it (S.F.); elenamaria.sala@irccs-sangerardo.it (E.S.); francesca.crosti@irccs-sangerardo.it (F.C.); leda.dalpra@unimib.it (L.D.); gaia.roversi@unimib.it (G.R.); giovanni.cazzaniga@irccs-sangerardo.it (G.C.); 2 School of Medicine and Surgery, University of Milano-Bicocca, 20900 Monza, Italy; serena.redaelli@unimib.it (S.R.); angela.bentivegna@unimib.it (A.B.); marialuisa.lavitrano@unimib.it (M.L.); 3 Department of Obstetrics, Fondazione IRCCS San Gerardo dei Tintori, 20900 Monza, Italy; sabrina.cozzolino@irccs-sangerardo.it (S.C.); maria.verderio@gmail.com (M.V.)

**Keywords:** prenatal diagnosis, genomic comparative hybridisation array, cytogenetic analysis, chromosomal translocation, genomic deletion, PEX3 gene

## Abstract

When an increased nuchal translucency (>3.00 mm) is observed during the echographic examination of a foetus in the first trimester of pregnancy, an increased risk of chromosomopathy is considered, and the pregnant woman is offered the possibility of an invasive investigation. Here, we focused our attention on prenatal diagnosis issues in cases of foetuses with cytogenetically balanced reciprocal translocations. We report the finding of a cytogenetically balanced, de facto genomically unbalanced translocation that poses a challenge in a case of prenatal diagnosis, changing the risk of Down syndrome in a Zellweger syndromic spectrum risk (*PEX3* deletion). At term, a healthy baby was born. This case teaches that prenatal diagnosis in cases of foetuses at increased risk of chromosomal abnormality imperatively requires molecular investigation in addition to a morphological karyotype.

When an increased nuchal translucency (>3.00 mm) is found in a foetus during the first trimester, even as the only clinical indication (isolated indication, alarming for the health condition of the foetus itself), the pregnant woman is offered the possibility of invasive investigation such as chorionic villus sampling to verify the foetal karyotype.

In the literature for these cases, numerous articles report the frequency of karyotype abnormalities, which is extremely variable between 0.5% and 6.6% [1]. Moreover, it is emphasised that the use of a comparative genomic hybridisation (CGH) array in foetuses with a normal karyotype detects on average an extra 5% of pathological copy number variations (CNVs). Normally, in these cases, a trisomy is expected, with trisomy 21 as the most frequent, but it is possible that structural abnormalities of the chromosomes, such as translocations or rare genetic/genomic conditions, are evidenced. Numerous genetic disorders have in fact been identified in association with the increase in nuchal translucency [2,3,4]; these include, for example, 22q11 micro-deletion syndrome, Noonan syndrome, and Smith–Lemli–Opitz syndrome [1].

We report the finding of a cytogenetically balanced, de facto genomically unbalanced translocation that poses a challenge in a case of prenatal diagnosis, changing the risk for Down syndrome in a Zellweger syndromic spectrum risk (Figure 1 and Figure 2).

CGH array analysis was also performed on both parents. The mother had a normal molecular karyotype, while the father showed a deletion in the same band of the cytogenetic translocation breakpoint (**D**). In all the consulted databases, no similar deletions were reported, so the CNV was considered as clinically uncertain (VOUS) because the foetal morphology was apparently normal and also because it was present in the molecular karyotype of the phenotypically normal father. The final foetal karyotype was as follows:

46,XY,t(3;6)(q25.3;q25.1)pat.arr[GRCh37] 6q24.2(143512294_144136217)x1 pat.

The lost genes in the deletion were the following: *AIG1* (exons from 4 to 6), *ADAT2*, *PEX3* (OMIM613164), *FUCA2*, *PHACTR2-AS1*, and *PHACTR2* (exons from 1 to 12).

To verify if the chromosome 6 alleles were biparentally inherited, thus excluding the uniparental disomy of chromosome 6 that is associated with transient neonatal diabetes mellitus [5,6], six microsatellites mapped in 6q13-q26 were tested. The analysis showed evidence of biparental allelic heredity.

The pregnancy progressed normally, and the foetus underwent obstetric ultrasound scans every 4-6 weeks that consistently documented regular foetal anatomy and foetal biometry at the upper limits, typical in the third trimester. The baby was born at 38+2 gestational weeks via caesarean section for breech presentation. The parameters at birth were as follows: Apgar at 1′ and 5′ minute: 8 and 9; weight: 3330 g; length: 49 cm (32nd percentile); CC: 35.5 cm (80th percentile). Nothing was reported at the first paediatric visit. After 18 months of life, the baby was in perfect health and the mother reported that they are very bright.

It is known that an increased thickness of the foetal nuchal fold is an “indicator” of risk for chromosomal/genetic abnormality [1,3]. Hence, following counselling, the mother was given the option to perform a placenta biopsy and both morphological and molecular karyotyping. This is because it has been established that the CGH array adds approximately 5% of pathological variants in the presence of a normal chromosomal karyotype [1]. In the case presented here, the biochemical test indicated both a particularly increased risk of numerical chromosomal abnormalities and an increase in nuchal translucency. The result of the genetic investigations showed a translocation between chromosome 3 and chromosome 6 of paternal origin, associated with a molecular deletion containing the *PEX3* gene.

De Gregori et al. described cryptic genomic abnormalities in 59 patients showing clinical phenotypes and a balanced karyotype [7]. Moreover, phenotypically normal individuals showing an apparently balanced translocation, at deep analysis with CGH array, were found to have an imbalance with gene disruption [8].

*PEX3* (OMIM613164) gene maps in 6q24.2 and the genomic coordinates are from nt 143,771,942 to nt 143,811,753 (GRCh37 assembly). In 6q24.2 micro-deletion, the *PEX3* gene is completely included, so one copy of the gene is lost. At the extremities of the deletion, the two interrupted genes (*AIG1* and *PHACTR2*) are not associated with pathology and are not dose-sensitive. The mutations in the *PEX3* gene are associated with disease risk in an autosomal recessive manner and *PEX3* is a moderately dose-sensitive gene.

In Decipher Database, the *PEX3* haploinsufficiency score (HI) is 27.6% (values from 0 to 10% mean “more likely haploinsufficient gene”, from 90 to 100% “more likely to not exhibit haploinsufficiency”), while the probability of Loss-of-function (LOF) Intolerance (pLI) is 0.02 (values ≥ 0.9 LOF not tolerated, ≤0.1 LOF tolerated). These values collectively suggest that this gene does not appear to be haploinsufficient and that heterozygous mutations do not seem to have deleterious effects.

Peroxins are essential for peroxisome function, and genetic abnormalities that alter their biogenesis constitute an autosomal recessive heterogeneous group of diseases known as Zellweger spectrum disorders (ZSD), characterised by the absence or reduction in functional peroxisomes in cells. Due to the phenotypic variability, ZSD was originally described as multiple distinct syndromes including Zellweger syndrome (ZS), neonatal adrenoleukodystrophy (NALD), infantile Refsum disease (IRD), rhizomelic chondrodysplasia punctata type 1 (RCDP1), and Heimler syndrome [9,10,11]. Based on a common peroxisomal basis, these disorders are now identified as ZDS, ranging from severe, intermediate, and milder phenotypes [10].

Disease severity often coincides with the age at which symptoms first appear [12]. Neonatal presentation is associated with a severe phenotype, with hypotonia, reduced spontaneous movements, feeding problems, seizures, direct hyperbilirubinemia, and elevated liver enzymes. Facial dysmorphism, large fontanelles, wide sutures, hypoplastic supraorbital ridges, and a broad nasal bridge are often reported. Ocular abnormalities include glaucoma, cataracts, and retinopathy. Death generally occurs within the first year of life [9,13].

Childhood presentation is associated with developmental delay, failure to thrive, retinal dystrophy, sensorineural hearing loss, feeding problems, hepatic dysfunction, and adrenal insufficiency. A regression of previously attained neurological milestones can occur secondary to demyelination (progressive leukodystrophy). Death may occur prior to adolescence [9,13].

In the adolescent and adult presentation of mild or absent developmental delay, neuroregression, cerebellar ataxia, peripheral neuropathy, adrenal insufficiency, and leukodystrophy can be observed. Life expectancy is variable [9,13].

The clinical spectrum of ZSD is due to mutations in one of the 13 *PEX* genes encoding peroxins. Pathogenic mutations in *PEX1* are responsible for about 60% of ZS cases, while the literature shows that homozygous pathogenic variants in the *PEX3* gene are responsible for only 0.7%; however, there are no data on deletions and/or duplications of the gene [14].

Since *PEX3* is involved in less than 1% of cases, and considering an incidence of ZS of 1/133000 (NY State newborn screening), the risk that the described foetus may be affected by ZS depends on the probability that the mother is a healthy carrier of a mutation in the same gene. The calculation of the healthy carrier in the general population for *PEX3* mutation is about 1 in 1820; therefore, the theoretical risk of ZS for the foetus, which is already a carrier of the paternal *PEX3* deletion, is approximately 1 in 3640 (0.03%).

Generally, prenatal diagnosis for ZSD is feasible if parental variants are known; a carrier test for *PEX3* mutations is technically feasible, but gene sequencing is burdened with too many variants of unknown significance, and is not clinically actionable, especially in prenatal context. Otherwise, biochemical screening assays in body fluids, i.e., amniotic, could be performed, but with limited diagnostic sensitivity. Since the “a priori” risk of the affected foetus was very low, the foetal development was normal with normal morphology at ultrasound examination, and, not in the least due to the high risk for chromosomal unbalanced conceptions due to an advanced maternal age and paternal translocation, the parents decided to continue the pregnancy. No foetal anomalies were evidenced at accurate ultrasound follow up and a healthy baby was born at term. No genetic tests were suggested for the reproductive risk related to Zellweger syndrome because the mother was a known carrier of the *BRCA2* mutation and underwent ovariectomy.

In conclusion, this case teaches that prenatal diagnosis in cases of foetuses at an increased risk of chromosomal abnormality imperatively requires molecular investigation, as does the morphological karyotype. Both complement each other, with one showing structural abnormalities and the other highlighting the loss and/or duplication of regions or individual gene anomalies below the level of resolution of classical cytogenetics.

## Figures and Tables

**Figure 1:**
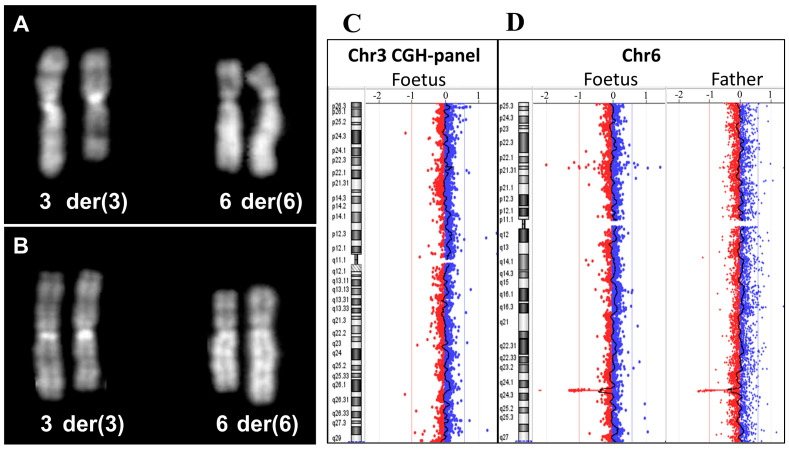
A 42-year-old G3P0 pregnant woman (2 first trimester miscarriages) was seeking prenatal diagnosis due to advanced age. On the first ultrasound, there was an increased nuchal translucency, 3.2 mm, while the general development was in the normal range. The combined test (PAPP-A and free beta-HCG) resulted in a high risk for trisomy 21, 1:5, and trisomy 13, 1:62. A chorionic villus sampling was performed at 12.5 weeks gestational age from cytotrophoblasts and after cultures of mesenchymal tissue; a male karyotype was observed with an apparently balanced translocation involving chromosomes 3 and 6 (**A**). The extension of the chromosomal analysis to parents showed the same translocation in the paternal karyotype and allowed us to define the breakpoints in 3q25.1 and 6q24.2 (**B**). The maternal karyotype was normal. At the same time, a CGH array was performed during a placental biopsy and deletion was evident: arr[GRCh37] 6q24.2(143512294_144136217,)x1 (**C**,**D**). The deletion was heterozygous, at a size of 624 Kb of chromosome 6 at cytoband q24.2, and it included the OMIM gene *PEX3*.

**Figure 2:**
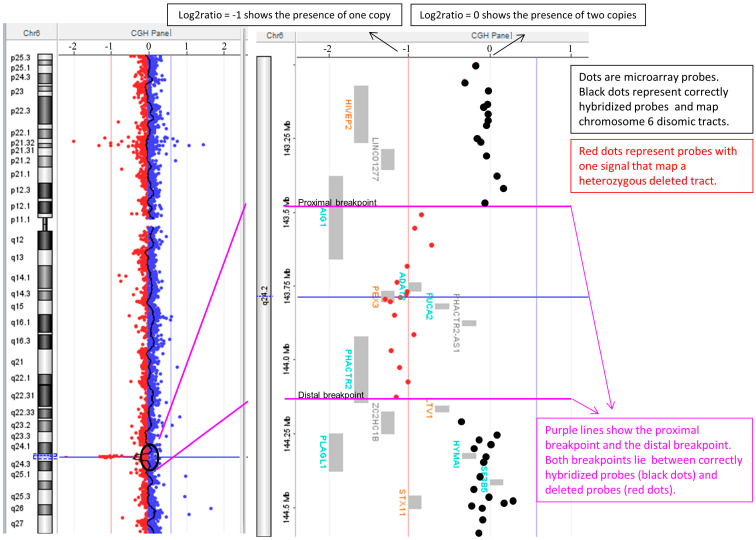
In our case, it could be hypothesised that the mechanism that produced the translocation also produced the micro-deletion in the genome; in fact, the break point of the micro-deletion maps in the cytogenetic band 6q24, according to the CGH array results.

## Data Availability

The authors declare that the data for this research are available from the correspondence authors upon reasonable request.
